# Transplantation of Gut Microbiota From High-Fat-Diet-Tolerant Cynomolgus Monkeys Alleviates Hyperlipidemia and Hepatic Steatosis in Rats

**DOI:** 10.3389/fmicb.2022.876043

**Published:** 2022-03-25

**Authors:** Jiang-Mei Gao, Jun-Hua Rao, Zhi-Yuan Wei, Shou-Yue Xia, Li Huang, Ming-Tian Tang, Geoff Hide, Ting-Ting Zheng, Jia-Huan Li, Guo-An Zhao, Yun-Xiao Sun, Jian-Huan Chen

**Affiliations:** ^1^Guangdong Key Laboratory of Animal Conservation and Resource Utilization, Guangdong Public Laboratory of Wild Animal Conservation and Utilization, Institute of Zoology, Guangdong Academy of Sciences, Guangzhou, China; ^2^Joint Primate Research Center for Chronic Diseases, Jiangnan University and Institute of Zoology, Guangdong Academy of Sciences, Guangzhou, China; ^3^Laboratory of Genomic and Precision Medicine, Wuxi School of Medicine, Jiangnan University, Wuxi, China; ^4^Biomedical Research Centre and Ecosystems and Environment Research Centre, School of Science, Engineering and Environment, University of Salford, Salford, United Kingdom

**Keywords:** cynomolgus monkey, hyperlipidemia, high-fat diet, fecal microbiota transplantation, *Megasphaera*

## Abstract

Emerging evidence has been reported to support the involvement of the gut microbiota in the host’s blood lipid and hyperlipidemia (HLP). However, there remains unexplained variation in the host’s blood lipid phenotype. Herein a nonhuman primate HLP model was established in cynomolgus monkeys fed a high-fat diet (HFD) for 19 months. At month 19%, 60% (3/5) of the HFD monkeys developed HLP, but surprisingly 40% of them (2/5) exhibited strong tolerance to the HFD (HFD-T) with their blood lipid profiles returning to normal levels. Metagenomic analysis was used to investigate the compositional changes in the gut microbiota in these monkeys. Furthermore, the relative abundance of *Megasphaera* remarkably increased and became the dominant gut microbe in HFD-T monkeys. A validation experiment showed that transplantation of fecal microbiota from HFD-T monkeys reduced the blood lipid levels and hepatic steatosis in HLP rats. Furthermore, the relative abundance of *Megasphaera* significantly increased in rats receiving transplantation, confirming the successful colonization of the microbe in the host and its correlation with the change of the host’s blood lipid profiles. Our results thus suggested a potentially pivotal lipid-lowering role of *Megasphaera* in the gut microbiota, which could contribute to the variation in the host’s blood lipid phenotype.

## Introduction

Hyperlipidemia (HLP) is considered one of the main contributing factors in developing metabolic syndromes and increasing the risk of atherosclerosis, stroke, coronary heart disease, and myocardial infarction (Karr, 2017; Panahi et al., 2018; Libby et al., 2019). In addition, high blood cholesterol (total cholesterol, TC) is a strong risk factor for hypertension, fatty liver, and diabetes (Elkins and Friedrich, 2018).

Recently, emerging evidence suggests the role of gut microbiota in human health and disease (Nicholson et al., 2012; Qin et al., 2012; Guo et al., 2016; Tong et al., 2018). Gut dysbiosis, defined as an imbalance in gut microbial communities, has been linked to a variety of diseases, including malnutrition, inflammatory bowel disease, neurological disorders, and cancer, as well as diabetes and obesity (Blanton et al., 2016; DeGruttola et al., 2016). For example, in genetically obese (ob/ob) mice, compared with wild-type and heterozygous lean counterparts, the abundance of Firmicutes was significantly increased and Bacteroidetes were decreased (Turnbaugh et al., 2006, 2009).

Probiotics could confer health benefits on the host by protecting them from gut dysbiosis. *Akkermansia muciniphila* is currently recommended as a new potential complementary therapy for obesity, diabetes, and liver diseases in clinics (Cani and de Vos, 2017; Plovier et al., 2017). It has been demonstrated that there are metabolic benefits of *Parabacteroides distasonis* (*P. distasonis*) on decreasing weight gain, hyperglycemia, and hepatic steatosis in ob/ob and high-fat diet (HFD)-fed mice. Administration of *P. distasonis* into obese mice dramatically changed the profile of secondary bile acids (Wang et al., 2019). In addition, *Faecalibacterium prausnitzii* and the peptides secreted by it showed anti-inflammatory effects on chemically induced colitis in mice (Breyner et al., 2017). *Lactobacillus* was reported to have a lipid-lowing effect on hypercholesterolemia and hyperlipidemic rat or mice (Zhou et al., 2013; Singh et al., 2015; Jiang et al., 2019; Qian et al., 2019). Therefore, it is promising to improve such diseases and gut dysbiosis by targeting the gut microbiota using probiotics.

As a typical chronic disease, HLP is closely related to gut microbiota dysbiosis and could also be potentially alleviated and cured through gut microbiota regulation. Given the high similarity to humans in terms of genetics, anatomy, reproduction, development, and metabolism, non-human primates (NHPs) are used as distinctive and indispensable model organisms in various areas of biomedical research and disease studies (Manara et al., 2019). Furthermore, it has been found that NHPs in captivity have a similar gut microbiota composition to that in humans (Clayton et al., 2016).

To understand the role of the gut microbiota in HLP, we used NHPs (cynomolgus monkeys) fed a high-fat diet (HFD) to establish an HLP model in the current study. HFD monkeys exhibited HLP by Month 7. After 12 months of HFD feeding, some individuals of the monkeys developed tolerance to HFD with their blood lipid profiles returning to normal levels. To explore the dynamics of the gut microbiota associated with such changes in lipid profiles, we conducted metagenomic sequencing in the HFD monkeys. Furthermore, transplantation of fecal microbiota from HFD-tolerant cynomolgus monkeys alleviated HLP and hepatocyte lesion in HFD rats.

## Materials and Methods

### Animals

Ten male cynomolgus monkeys were purchased from Guangdong Landau Biotechnology Co. Ltd. (Guangzhou, China) which is accredited by the Association for the Assessment and Accreditation of Laboratory Animal Care International (AAALAC). All the animals were confirmed to be in healthy condition by records and veterinary examination before the experiment.

All monkeys were kept in a well-controlled and comfortable environment with temperature (16°C–28°C) and relative humidity of 40%–70%, as well as a 12/12-h light–dark cycle. All the animals were free to access food and drinking water. The protocol for this study was approved by the Institutional Animal Care and Use Committee of Guangdong Landau Biotechnology Co., Ltd. (Code: LD20150518).

All monkeys were randomly divided into two groups, including an HFD group (*n* = 5) and a normal chow diet group (NCD, *n* = 5). The NCD group was fed normal chow, while the HFD group was fed normal chow daily plus emulsion containing 10% sucrose, 10% lard, 1% cholesterol, and 0.5% cholate (5 ml/kg body weight) *via* nasogastric gavage 6 days a week. All the monkeys in the HFD group were fed HFD for 19 months.

Twenty seven male Sprague–Dawley (SD) rats used for validation experiments were purchased from Guangzhou University of Chinese Medicine. All rats were kept in a specific pathogen-free environment with free access to food and water. The protocol for this study was approved by Institute of Zoology, Guangdong Academy of Sciences (GIABR20200908).

The rats were randomly divided into three groups (*n* = 9 for each group): an NCD group (NCD), an HFD group, and a fecal microbiota transplantation group (HFD + M). All rats were fed with an NCD. The HFD and HFD + M groups were fed normal chow daily plus the same emulsion used in the monkeys (5 ml/kg body weight) by gavage (six days a week for 12 weeks.

### Fecal Microbiota Transplantation

Fresh stool samples from monkeys that showed tolerance to the HFD were pooled, suspended in sterile phosphate buffer saline (PBS, pH = 7.0), and centrifuged at 500 *g* for 5 min. 200 μl of the prepared supernatant (10^8^ CFU) was given to the HFD + M group of rats *via* oral gavage 6 days per week at week 7–12 of HFD feeding.

### Biochemical Analysis

Blood samples of rats and monkeys were periodically collected from the ocular vein and the upper limb saphenous vein, respectively. Serum total cholesterol (TC), triacylglycerol (TG), low-density lipoprotein cholesterol (LDL-C), and high-density lipoprotein cholesterol (HDL-C) were measured using commercially available kits from Guangdong Lewwin Pharmaceutical Research Institute Co., Ltd. (Guangzhou, China).

### Histological Analysis

Liver tissues of rats were collected, fixed in 4% paraformaldehyde, paraffin-embedded, and sectioned at 5 μm. Hematoxylin and eosin (H&E) and Oil Red O staining of paraffin sections were conducted following a standard method.

### Metagenomic Sequencing and Analysis

DNA of macaque fecal samples was collected at month 19 of HFD feeding and sequenced on the HiSeq-X10 platform (Illumina) using a paired-end 150 bp configuration. Cleaning data were obtained by filtering raw data using KneadData (v0.7.4).^1^ Karaken2 (v2.0.8) was used for taxonomic analysis (Wood and Lu, 2019), and vegan 2.5–7 was used for diversity indices analysis.

### 16S rRNA Gene Sequencing and Analysis

SD rat fecal samples were collected at Week 12 for gut microbial analysis. Bacterial genomic DNA was extracted from frozen fecal samples stored at −80°C using TIANamp Stool DNA kit (Cat.#DP328, Tiangen, China) according to the manufacturer’s instructions.

The hypervariable V4 regions of bacterial 16 s rRNA genes were amplified using the polymerase chain reaction and V4-specific primers as described previously (Wei et al., 2021). After quality control, PCR products were purified and sequenced on an Ion S5XL sequencer (Thermo Fisher Scientific, Waltham, Massachusetts) with a single-end 400-bp read length configuration.

Bioinformatic analysis of the 16S rRNA gene sequencing data was conducted using the QIIME2 (version 2018.6.0) analysis pipeline (Bolyen et al., 2019). Briefly, the dada2 program was used to filter and remove low-quality and chimeric sequences, and generate unique feature tables equivalent to operational taxonomic unit (OTU) tables at exact match or 100% sequence similarity. Taxonomic identifications were then assigned to these features using the q2-feature-classifier and the full-length SILVA database (release r138) at a 99% similarity cutoff (Parks et al., 2014). PICRUSt (version 1.1.4) was used to predict microbial functions from the 16S rRNA gene sequencing data, which were further categorized using the BRITE hierarchy of the KEGG database (Langille et al., 2013). Differences in the microbial functions were then analyzed by the Linear discriminant analysis (LDA) Effect Size (LEfSe) algorithm with a log (LDA) score cutoff of 2 and default settings (Segata et al., 2011).

### Statistical Analysis

Statistical analysis was performed using GraphPad Prism V.7.0a (GraphPad Software, United States) and the R statistical language (version 3.6.0). The levels of serum lipid, the relative abundance of OTUs, and alpha diversity indices among groups were compared using the *Student’s t*-test and evaluated for pair-wise inter-group differences with Tukey’s *post-hoc* test if overall significance was found.

## Results

### Tolerance to HFD in Monkeys After Long-Term Dietary Induction

**Figure 1:**
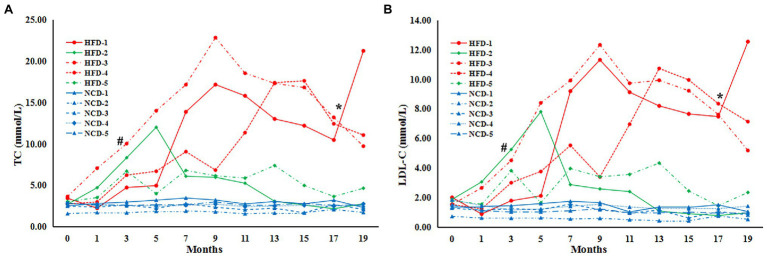
Serum lipids in cynomolgus monkeys during HFD feeding. Serum levels (mmol/L) are shown for **(A)** total cholesterol (TC), **(B)** low-density lipoprotein cholesterol (LDL-C). HFD: high-fat diet monkeys; NCD: normal chow diet. Significant differences are indicated as ^#^, at month 3, HFD monkeys *vs* NCD monkeys, *p* < 0.05; *, at Month 17, HFD-1/HFD-3/HFD-4 (HFD-intolerant) monkeys *vs* HFD-2/HFD-5 (HFD-tolerant) monkeys, *p* < 0.05.

To explore the characteristics of HLP, the levels of TC and LDL-C were significantly higher in monkeys fed HFD than NCD controls (*n* = 5) by 3 months (TC, HFD *vs* NCD, *p* = 0.001; LDL-C, HFD vs. NCD, *p* = 0.003) (Figures 1A,B). After 7 months, all HFD monkeys showed HLP (TC > 6.20 mmol/L and LDL-C > 3.64 mmol/L) with higher levels of TC and LDL-C compared to the NCD controls (TC, HFD vs. NCD, *p* = 0.006; LDL-C, HFD vs. NCD, *p* = 0.007). Intriguingly, after 17 months of diet induction, 2 monkeys (HFD-2 and HFD-5) exhibited obvious tolerance to HFD with their blood lipid profiles returning to normal levels (TC, HFD-2/5 vs. NCD control, *p* > 0.05; HFD-2/5 vs. HFD-1/3/4, *p* = 0.004. LDL-C, HFD-2/5 vs. NCD control, *p* > 0.05; HFD-2/5 vs. HFD-1/3/4, *p* < 0.0001). In the subsequent analysis, we refer to HFD-2 and HFD-5 as HFD-tolerant (HFD-T), and HFD-1, HFD-3, and HFD-4 as HFD-intolerant (HFD-I). However, long-term HFD did not cause significant body weight changes in HFD monkeys compared to NCD monkeys by month 19 (data not shown).

### Gut Microbiota Profiles Associated With HFD Tolerance in Monkeys

**Figure 2:**
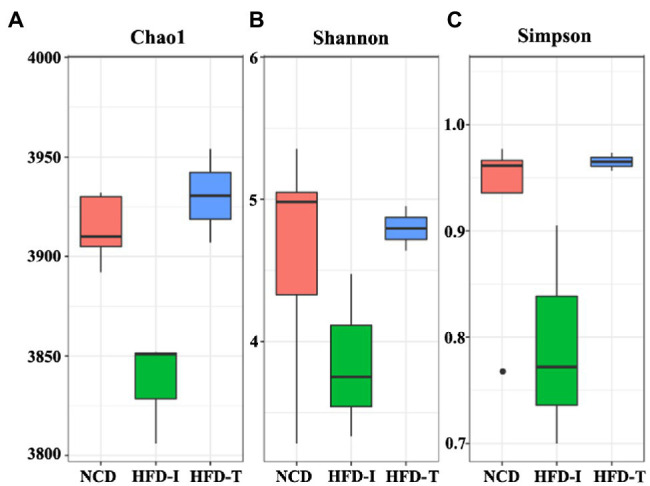
Boxplots showing changes in alpha diversity of the gut microbiota in NCD, HFD-I, and HFD-T monkeys. Alpha diversity indices including Chao1 **(A)**, Shannon’s **(B)** index, and Simpson’s index **(C)** are shown.

To explore the dynamics of gut microbiota associated with such changes in lipid profiles, we conducted metagenomic sequencing using fecal DNA from both HFD-T and HFD-I monkeys after HFD-induction for 19 months. Our results showed that long-term HFD induction changes the composition of the gut microbiota and slightly reduced alpha diversity indices (Chao1, *Shanno’s* index, and *Simpson*’s index) in HFD-I monkeys, while HFD-T monkeys had similar alpha diversity compared to NCD controls (Figure 2).

Analysis at the phylum level showed that HFD markedly decreased the relative abundance of Firmicutes, and increased the relative abundance of Bacteroidetes. In addition, Spirochaetes was increased in HFD-I monkeys. However, the relative abundance of Firmicutes was found to be partially increased in HFD-T monkeys, and Bacteroidetes also increased in HFD-I monkeys (Supplementary Figure S1).

**Figure 3:**
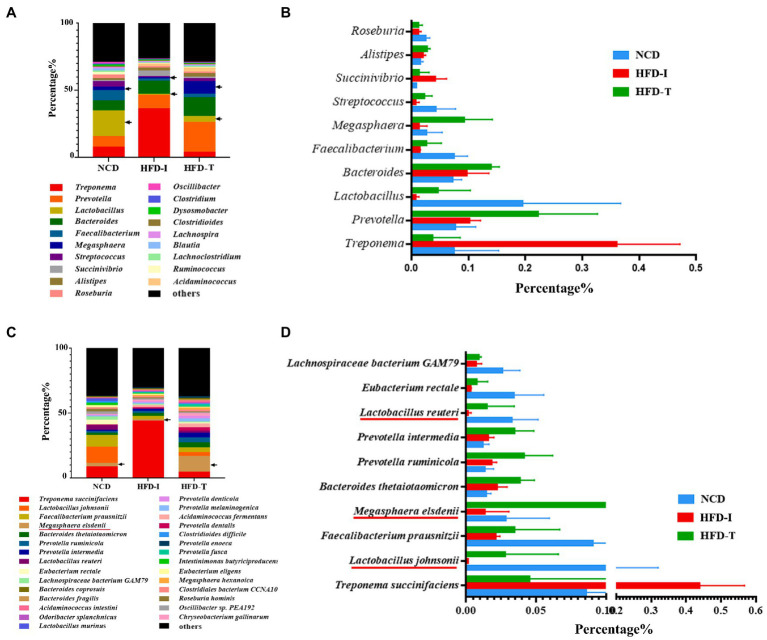
The most abundant genera and species in the gut microbiota of NCD, HFD-I, and HFD-T monkeys. Relative abundances of the gut microbiota at the genus level **(A)**, top 10 abundant genera **(B)**, relative abundances of the gut microbiota at the species level **(C)**, and top 10 abundant species **(D)** are shown.

A detailed analysis of the bacterial genera detected in each group is shown in Figure 3A. The relative abundance of *Lactobacillus* was the highest in NCD monkeys but was markedly reduced in HFD monkeys (HFD-I and HFD-T groups). Intriguingly, the relative abundance of *Prevotella*, *Bacteroides, Lactobacillus,* and especially *Megasphaera* were increased in HFD-T monkeys (Figures 3A,B).

**Figure 4:**
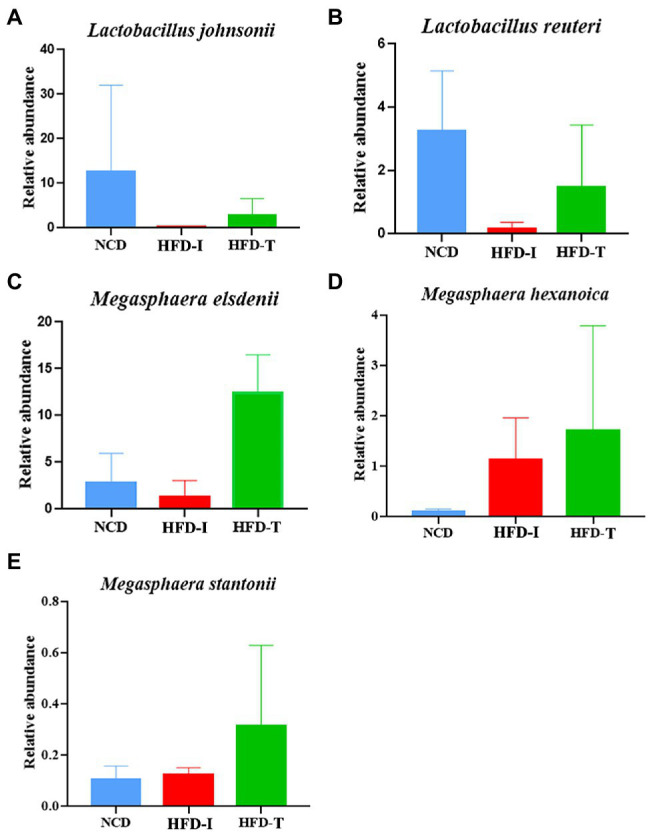
The relative abundance of selected species in the gut microbiota in NCD, HFD-I, and HFD-T monkeys. *Lactobacillus johnsonii* **(A)**, *Lactobacillus reuteri* **(B)**, *Megasphaera elsdenii* **(C)**, *Megasphaera hexanoica* **(D)**, and *Megasphaera stantonii* **(E)**.

Further analysis at the species level showed similar results to those observed at the genus level. As shown in Figure 3C, the relative abundance of *Lactobacillus johnsonii*, *Lactobacillus reuteri* and *Megasphaera elsdenii* were highest in NCD monkeys. However, the relative abundance of these bacteria was markedly reduced after long-term HFD feeding (Figure 3D). However, the relative abundance of *L. johnsonii* and *L. reuteri* were found to be partially increased in HFD-T monkeys (Figures 3D, 4A,B). However, *M. elsdenii* was dramatically increased and became predominant in HFD-T monkeys (Figures 3C,D, 4C). In addition, another two species of *Megasphaera* (including *Megasphaera hexanoica* and *Megasphaera stantonii*) were also increased in HFD-T monkeys (Figures 4D,E).

### Transplantation of Fecal Microbiota From HFD-T Monkeys to HFD Rats Alleviated HLP and Hepatic Steatosis

**Figure 5:**
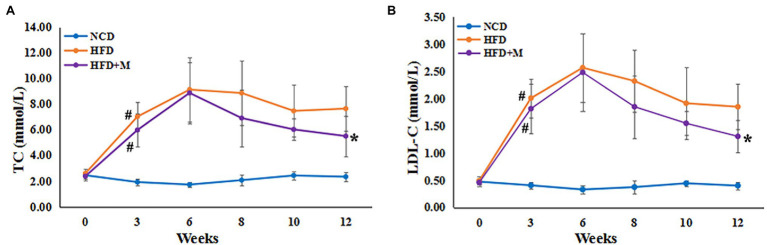
Serum lipids in SD rats following fecal microbiota transplantation from HFD-T monkeys. Serum levels (mmol/L) are shown for **(A)** total cholesterol (TC), **(B)** low-density lipoprotein cholesterol (LDL-C). NCD, Normal Chow Diet rats; HFD, HFD rats; HFD + M, HFD rats with transplantation from the fecal microbiota from HFD-T monkeys. ^#^, at Week 3 of HFD feeding, HFD and HFD + M rats *vs* NCD rats, *p* < 0.05; *, at Week 12, HFD rats *vs* HFD + M rats, *p* < 0.05.

**Figure 6:**
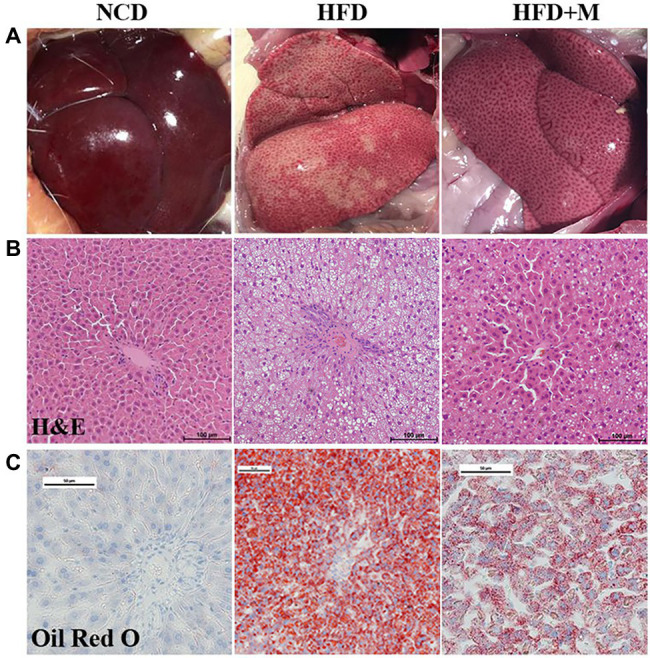
Changes in hepatic steatosis after fecal microbiota transplantation. **(A)** Photos showing liver morphology. **(B)** H and E staining of liver section. Scale bars, 100 μm. **(C)** Liver Oil Red O staining. NCD, Normal Chow Diet rats; HFD, HFD rats; HFD + M, HFD rats received fecal microbiota transplant from HFD-T monkeys. Scale bars, 50 μm.

To validate the possible lipid-lowering effects of the gut microbiota in HFD-T monkeys, fecal microbiota from HFD-T monkeys was transplanted to HLP rats fed with an HFD. All rats showed significantly higher levels of TC and LDL-C compared to NCD rats after 3 weeks of HFD feeding. Transplantation of fecal microbiota from HFD-T monkeys alleviate HLP in the recipient HFD rats with significantly lower levels of TC (NCD *vs* HFD, *p* < 0.0001; NCD *vs* HFD + M, *p* < 0.0001; HFD *vs* HFD + M, *p* = 0.0227) and LDL-C (NCD *vs* HFD, *p* < 0.0001; NCD *vs* HFD + M, *p* < 0.0001; *p* = 0.0094; Figures 5A,B). Furthermore, HFD-fed rats showed enhanced lipid accumulation, which was markedly inhibited with improved hepatic steatosis by the fecal microbiota transplantation in the HFD + M mice (Figure 6).

Fecal Microbiota Transplantation Changed Gut Microbiota of HFD Rats.

**Figure 7:**
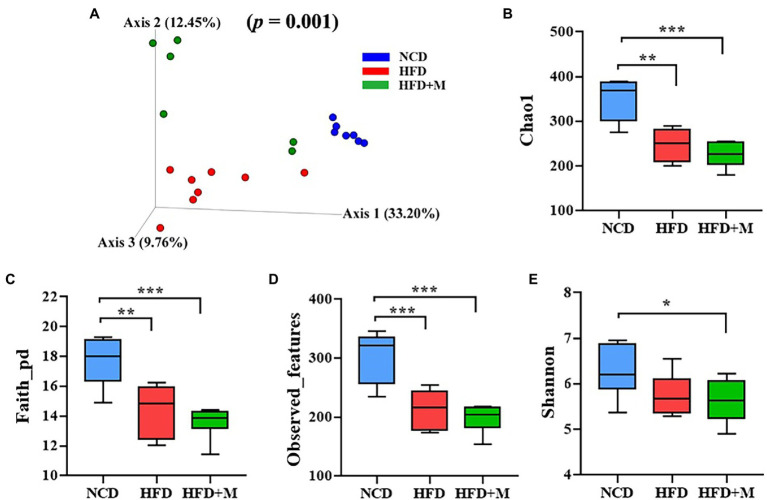
The beta diversity, structure, and richness of the gut microbiota in three groups of rats. **(A)** Nonmetric multidimensional scaling (NMDS) score plot based on principal component analysis (PCA) score plot based on microbiota composition. **(B–E)** The gut microbiota diversity as calculated using Shannon’s index **(B)**, Chao1 **(C)**, Faith-pd **(D)**, and observed-features **(E)** index of each rat group: NCD, Normal Chow Diet (control) rats; HFD, HFD rats; HFD + M, HFD rats received fecal microbiota transplant from HFD-T monkeys. Values are presented as mean ± SE. (*n* = 7). *, *p* < 0.05; **, *p* < 0.01; ***, *p* < 0.001, compared with NCD rats.

To further investigate the lipid-lowering mechanism of the gut microbiota from the HFD-T monkeys, we also analyzed the gut microbiota composition of rats in the NCD, HFD, and HFD + M groups using 16S rRNA gene high-throughput sequencing. Principal component analysis (PCA), the NCD, HFD, and HFD + M groups of rats each presented a separated clustering of microbiota composition (Figure 7A). Such results demonstrate that transplantation of fecal microbiota significantly changed the gut microbiota profiles in rats (*p* = 0.001).

In addition, it has been demonstrated that HFD could significantly reduce the richness and diversity of the gut microbiota in HFD fed rats (including both the HFD and HFD + M groups) by using a range of alpha diversity indices (Shannon, Chao1, Faith-pd, and Observed-features; Figures 7B–E). However, there was no significant difference in the richness and alpha diversity of the gut microbiota between the HFD and HFD + M groups (Figures 7B–E).

As shown in Supplementary Figure S2, at the phylum level analysis HFD significantly decreased the relative abundance of Firmicutes and increased the relative abundance of Bacteroidetes. These changes are consistent with findings in monkeys. However, the relative abundance of Firmicutes and Bacteroidetes in HFD + M rats showed no significant changes compared to HFD rats.

**Figure 8:**
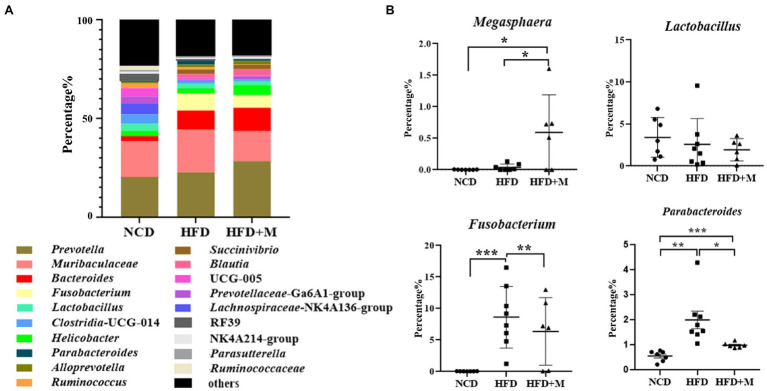
Relative abundances of gut microbiota at genus level in different rat groups. **(A)** The most abundant genera (top 20) of the gut microbiota in SD rats of different groups. **(B)** The relative abundance of *Megasphaera*, *Lactobacillus*, *Parabacteroides,* and *Fusobacterium* in fecal microbiota. NCD, Normal Chow Diet rats; HFD, HFD rats; HFD + M, HFD rats received fecal microbiota transplant from HFD-T monkeys. *, *p* < 0.05; **, *p* < 0.01; ***, *p* < 0.001.

Furthermore, we analyzed the differences in the gut microbiota at the genus level. Genera *Prevotella*, *Bacteroides,* and *Lactobacillus* were predominant in the rat gut microbiota and *Bacteroides* was significantly increased in HFD and HFD + M rats (NCD *vs* HFD, *p* < 0.001; NCD *vs* HFD + M, *p* < 0.0001; Figure 8A). However, *Fusobacterium* (NCD *vs* HFD, *p* < 0.001; HFD *vs* HFD + M, *p* < 0.01) and *Parabacteroides* (NCD *vs* HFD, *p* < 0.01; HFD vs. HFD + M, *p* < 0.01) were increased in HFD rats but were significantly decreased after fecal microbiota transplantation (Figure 8B). The abundance of *Lactobacillus* showed no significant difference among the three groups of rats (*p* > 0.05; Figure 8B). Interestingly, *Megasphaera* was significantly increased in HFD + M rats (*p* < 0.05), in line with its changes observed in HFD-T monkeys, implicating its role in the reduction of the host’s blood lipids.

**Figure 9:**
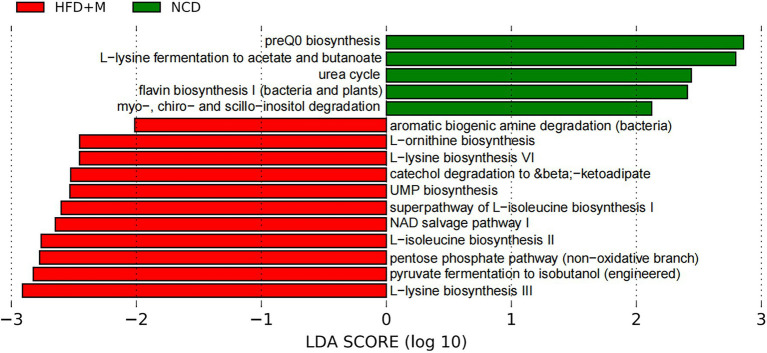
LEfSe analysis of microbial functions predicted by PICRUSt in different rat groups. NCD, Normal Chow Diet rats; HFD, HFD rats; HFD + M, HFD rats received fecal microbiota transplant from HFD-T monkeys; LDA, linear discriminant analysis (LDA); LEfSe, linear discriminant analysis effect size.

In addition, as shown in LEfSe analysis shown in Figure 9, PICRUSt prediction results indicated that transplantation of fecal microbiota from HFD-T monkeys changed various functions of gut microbiota in rats, particularly biosynthesis of amino acids, including L-lysine and L-isoleucine.

## Discussion

HLP is considered an important risk factor for cardiovascular diseases (CVDs). More than 17.9 million people died from CVDs in 2019, representing 32% of all deaths worldwide (Roth et al., 2020). However, emerging evidence shows that gut microbiota plays an important role the host’s energy metabolism and blood lipid level modulation (Velagapudi et al., 2010; Mestdagh et al., 2012). Therefore, we developed an NHP HLP model with high similarity to humans in genetics, physiology, and gut microbial composition, for analysis of the effect of gut microbiota on the host’s blood lipids. A unique and interesting feature of such an NHP model was that 40% of the monkeys in our study developed tolerance to HFD with normal lipid profiles after prolonged exposure. Such an NHP model provided a unique tool to investigate changes in the gut microbiota related to the development of HFD tolerance.

Previous studies have demonstrated that multiple species of *Lactobacillus* can reduce serum cholesterol, TG, and LDL-C, and have been used in clinical practice as potential probiotics (Zhou et al., 2013; Singh et al., 2015; Jiang et al., 2019; Qian et al., 2019). In the current study, we found that *Lactobacillus* (such as *Lactobacillus johnsoni* and *L. reuteri*) was significantly increased in HFD-T monkeys with TC and LDL-C returning to the normal level. However, after transplantation of fecal microbiota from HFD-T monkeys to HFD rats, *Lactobacillus* showed no significant change in HFD + M rats. Therefore, *Lactobacillus* might not be the major microbial microbe responsible for the reduction of serum lipids in the HFD + M rats.

In previous studies, *Parabacteroides* showed a beneficial effect against weight gain, hyperglycemia, hepatic steatosis, and HLP (Wang et al., 2019). However, the relative abundance of *Parabacteroides* was also significantly decreased in HFD + M rats. HFD rats showed the highest relative abundance of *Parabacteroides*, suggesting that *Parabacteroides* might not play a lipid-lowering effect role in HFD + M rats.

In our previous study, *Megasphaera* was found to be a dominant genus in the primate gut microbiota throughout different life stages and a key driver that contributes to long-term gut microbiota development (Wei et al., 2021). The genus belongs to the family *Veillonellaceae* under the order Veillonellales, class Negativicutes, and phylum Firmicutes (Maki and Looft, 2018). Previous studies have demonstrated that *M. elsdenii* could metabolize lactate and buffer fat and produce acetate, propionate, and butyrate (Chen and Wang, 2016; Chen et al., 2019). These short-chain fatty acids may act through down-regulating cholesterol biosynthesis and increasing the bile acid excretion to regulate the lipid levels (Illman et al., 1988; Marcil et al., 2003; Falcinelli et al., 2015, 2018). Our results found that *Megasphaera*, especially *M. elsdenii*, was a predominant component of the microbiota, and showed a dramatic increase in abundance in HFD-T monkeys. Surprisingly, the relative abundance of *Megasphaera* was also dramatically increased in HFD + M rats and this increasing trend was consistent with changes in the abundance of *Megasphaera* species in monkeys. In contrast, *Megasphaera* is almost undetectable in NCD and HFD rats without fecal microbiota transplantation. Therefore, it is reasonable to speculate that the *Megasphaera* (more likely *M. elsdenii*) successfully colonized in HFD + M rats after fecal microbiota transplantation, and *Megasphaera* might play a critical role in lowering blood lipids in these HLP animals.

Our results also indicated the functional impact of fecal microbiota transplantation from HFD-T monkeys on the gut microbiota in HFD rats, pointing to a potential role of amino acid biosynthesis. Effects of lysine and isoleucine on lipid metabolism have been implicated in previous studies. Dietary lysine restriction in rats was reported to cause lipid accumulation in skeletal muscle (Goda et al., 2021). Branched-chain amino acids leucine and isoleucine were found to reduce lipid accumulation in HFD-induced obese mice (Ma et al., 2020). It reminds a possible mechanism *via* altered biosynthesis of such amino acids in the gut microbiota in improving the host’s lipid metabolism.

Our current study demonstrates that changes in the gut microbiota are associated with variation of blood lipid phenotype in both monkeys and rats under HFD feeding. Transplantation of the gut microbiota from HFD-T monkeys alleviated HLP and hepatic steatosis in rats. Further studies are thus warranted to confirm the lipid-lowering and liver-protecting effects of *Megasphaera*, which could be potentially used as a probiotic to lower blood lipids, and improve hepatic steatosis in the treatment of HLP.

## Data Availability Statement

The datasets presented in this study can be found in online repositories. The names of the repository/repositories and accession number(s) can be found in the article/Supplementary Material.

## Ethics Statement

The animal study was reviewed and approved by the Institutional Animal Care and Use Committee of Guangdong Landau Biotechnology and Institute of Zoology, Guangdong Academy of Sciences.

## Author Contributions

J-MG, J-HR, Z-YW, and J-HC designed the study. J-MG, LH, M-TT, T-TZ, J-HL, G-AZ, and Y-XS conducted the experiments. J-MG, Z-YW, and S-YX conducted the data analysis data or performed the statistical analysis. J-MG, GH, J-HC, and J-HR drafted the manuscript. All authors contributed to the article and approved the submitted version.

## Funding

This study was supported in part by grants from the National Natural Science Foundation of China (no. 31671311), the Guangdong Key Laboratory of Non-human Primate Research (2020B121201006), GDAS’ Project of Science and Technology Development (2022GDASZH-2022010110), Guangdong Basic and Applied Basic Research Foundation (2019A1515012062 and 2020A1515010480), Natural Science Foundation of Guangdong Province (2018A030313307), the Program for High-Level Entrepreneurial and Innovative Talents Introduction of Jiangsu Province, the Taihu Lake Talent Plan, and Wuxi Institute of Translational Medicine.

## Conflict of Interest

The authors declare that the research was conducted in the absence of any commercial or financial relationships that could be construed as a potential conflict of interest.

## Publisher’s Note

All claims expressed in this article are solely those of the authors and do not necessarily represent those of their affiliated organizations, or those of the publisher, the editors and the reviewers. Any product that may be evaluated in this article, or claim that may be made by its manufacturer, is not guaranteed or endorsed by the publisher.

## Footnotes

^1^ https://huttenhower.sph.harvard.edu/kneaddata/
