# Supplementary figures and images for: Transplantation of Gut Microbiota From High-Fat-Diet-Tolerant Cynomolgus Monkeys Alleviates Hyperlipidemia and Hepatic Steatosis in Rats
Source: Front Microbiol. 2022 Mar 25;13:876043. doi: 10.3389/fmicb.2022.876043 (PMC8990751; doi:10.3389/fmicb.2022.876043)

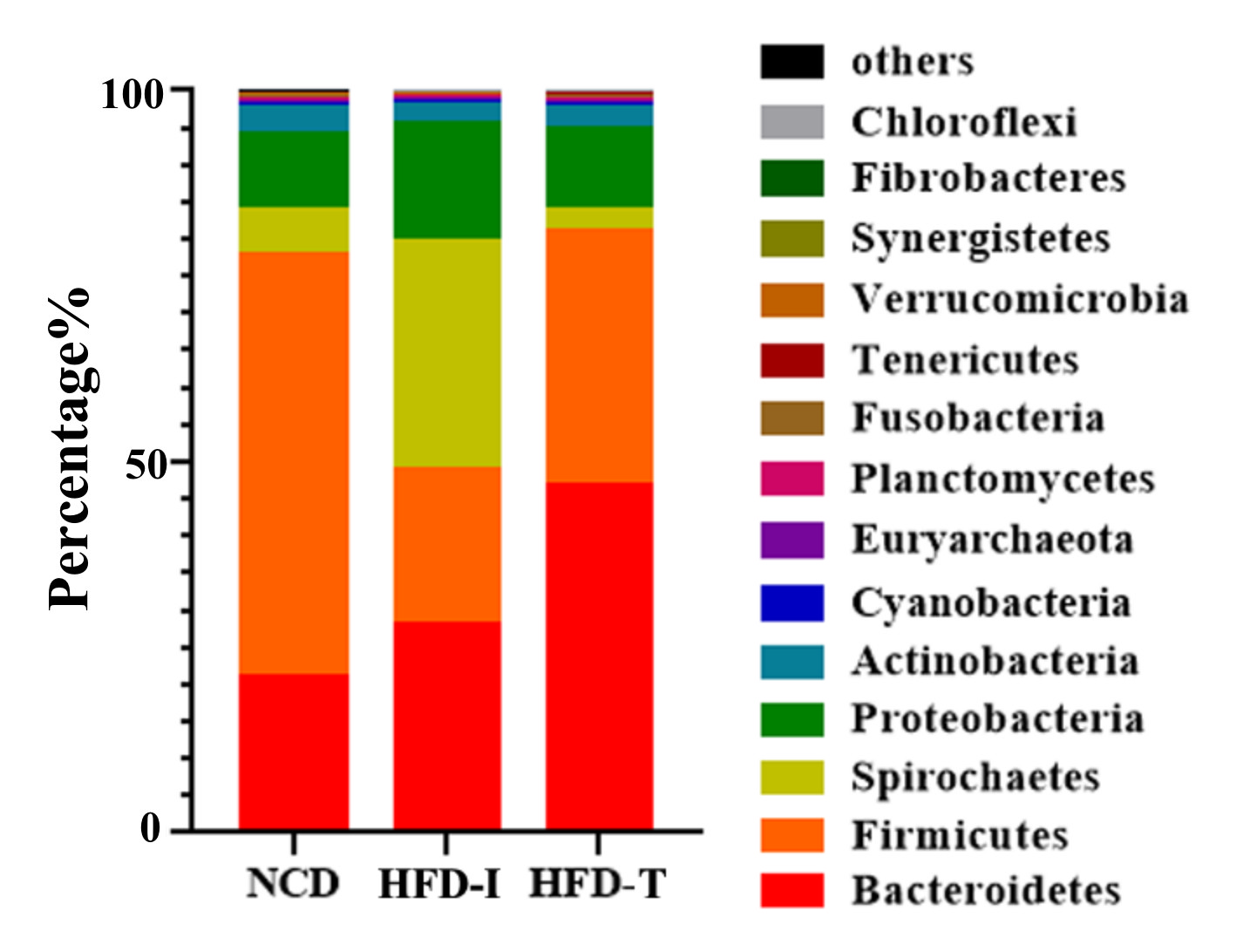

Supplement: Supplementary file 1 [file Image_1.tif]

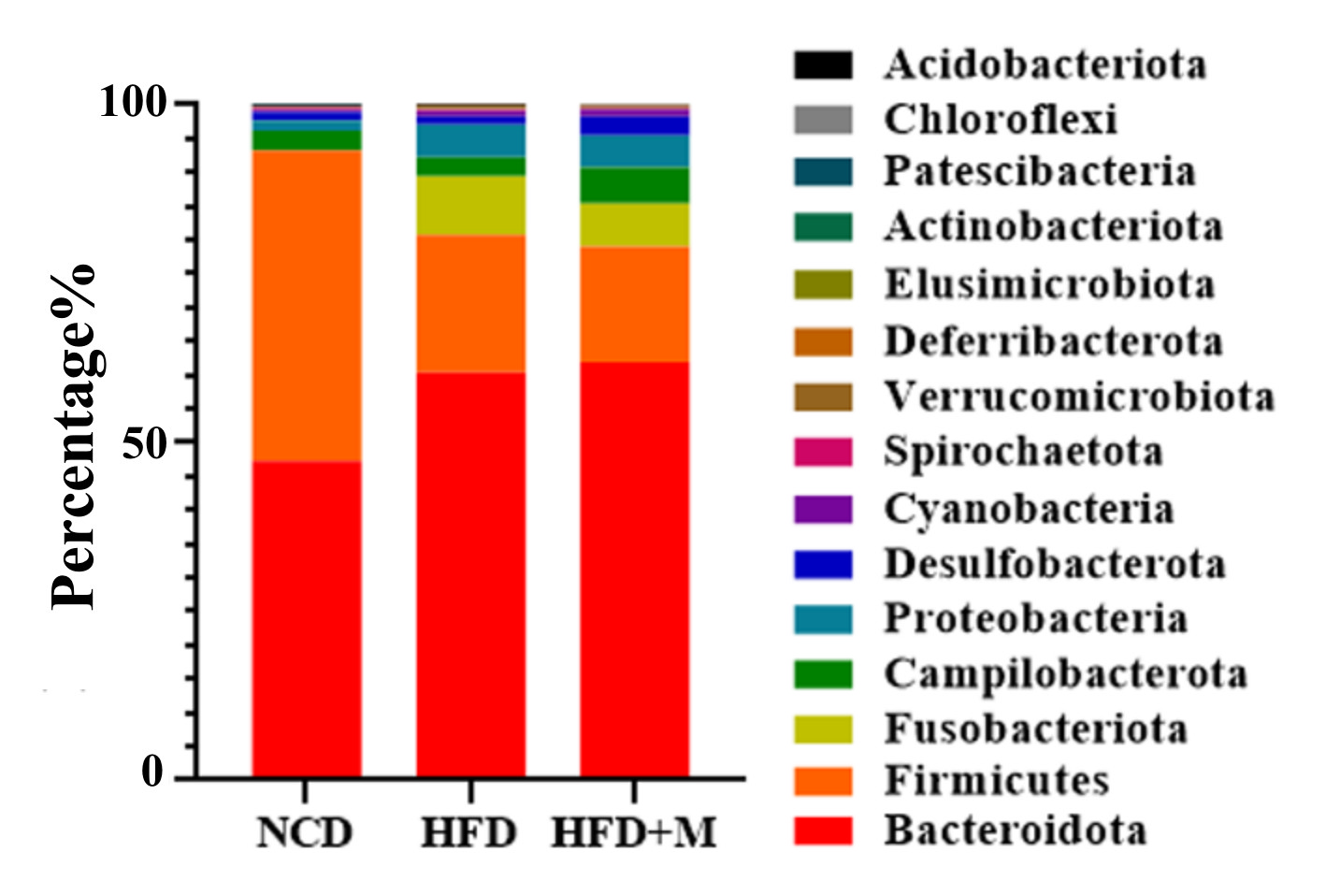

Supplement: Supplementary file 2 [file Image_2.tif]
